# Centrifugation-Based Purification Protocol Optimization Enhances Structural Preservation of Nucleopolyhedrovirus Budded Virion Envelopes

**DOI:** 10.3390/insects16040424

**Published:** 2025-04-17

**Authors:** Yong Pan, Jiming Yan, Yinong Zhang, Jiasheng Lin, Zhiquan Liang, Jingchen Sun

**Affiliations:** 1 Guangdong Provincial Key Laboratory of Agro-Animal Genomics and Molecular Breeding & Subtropical Sericulture and Mulberry Resources Protection and Safety Engineering Research Center, College of Animal Science, South China Agricultural University, Guangzhou 510642, China; panyongscau@gmail.com (Y.P.); gmyjm97@sina.com (J.Y.); yinongzh@foxmail.com (Y.Z.); linjs2025@163.com (J.L.); lzq0318@stu.scau.edu.cn (Z.L.); 2 College of Veterinary Medicine, South China Agricultural University, Guangzhou 510642, China

**Keywords:** baculovirus purification, viral envelope integrity, sucrose density gradient, cryo-EM, *Alphabaculovirus*, virion, AcMNPV

## Abstract

The structural integrity of viral envelopes is a critical determinant of infectivity for enveloped viruses, directly influencing vector stability, functional accuracy of surface-displayed epitopes, and preservation of native conformational states required for membrane protein studies. However, conventional purification methods often disrupt envelope integrity and cause envelope proteins to lose their activity. Here, we systematically compared discontinuous, continuous, and optimized continuous sucrose density gradient centrifugation protocols for purifying Autographa californica multiple nucleopolyhedrovirus (AcMNPV). Through cryo-EM, we demonstrated that our optimized continuous sucrose gradient protocol significantly increased the proportion of AcMNPV budded virions with intact envelopes from 36% to 81%, while preserving the metastable prefusion conformation of the fusion protein GP64. This advancement should prove useful for structural studies of viral envelope proteins and may enhance applications in gene therapy and vaccine development utilizing enveloped viruses.

## Simple Summary

Baculoviruses are widely used for scientific and medical applications, but purifying baculovirus virions without damaging their viral envelopes is a challenge. In this study, we compared the effects of three purification methods on the percentage of budded virions (BVs) with intact envelopes. Compared to the traditional protocols, the optimized continuous sucrose density gradient purification increased the percentage of intact viral envelopes from 36% to 81%. Cryo-electron microscopy (cryo-EM) confirmed preserved prefusion conformations of the envelope protein GP64. This advancement supports structural studies of envelope proteins and may improve applications in gene therapy and vaccine development using enveloped viruses.

## 1. Introduction

Viruses, as obligate intracellular parasites, largely depend on host cellular machinery to complete their replication cycle [1]. A critical initial step involves breaching the host plasma membrane barrier to deliver viral genetic material into the cellular interior [2,3]. This process typically requires interaction between viral structural components and host cell receptors, ultimately enabling translocation of the genome-containing capsid to appropriate intracellular sites for replication initiation [4,5]. Notably, enveloped viral species employ a sophisticated membrane fusion mechanism mediated by their bilipid envelope [6,7]. This biological orchestration depends crucially on two essential components: (1) structural integrity of the viral envelope, and (2) precisely coordinated functions of virally encoded transmembrane glycoproteins embedded within this membranous layer [8,9]. These fusion-competent glycoproteins mediate sequential conformational changes that drive thermodynamic merging of viral and host membranes [9,10]. The structural integrity of the viral envelope is indispensable not only as a core determinant of infectivity but also as a mechanistic basis for dissecting the structural–functional interplay of envelope-embedded glycoproteins during membrane fusion [11,12,13,14,15].

In contemporary biomedical research, viruses—once feared as pathogenic entities responsible for substantial historical mortality—underwent significant functional repurposing as molecular vectors [16,17,18,19]. These engineered virions now facilitate targeted gene delivery systems for transgenic applications, serve as therapeutic vectors in precision medicine applications, and function as antigen presentation platforms via surface-displayed epitopes [20,21,22,23]. In antiviral development, the low stability of most viral envelope proteins (e.g., HSV gB) makes it challenging to obtain their active prefusion structures, yet inhibitor design based on the prefusion conformation is critical for developing effective antiviral drugs [24,25,26,27]. The implementation of these biotechnological applications fundamentally depends on preserving the structural integrity of viral particles and the active conformation of the envelope protein during production workflows. Current viral purification methodologies frequently induce particulate degradation, particularly disrupting enveloped viruses with labile membrane architectures [28]. This operational limitation necessitates development of innovative fractionation techniques that maintain biophysical stability parameters. Such advancements hold particular urgency for advancing virus purification methods.

Baculoviruses are a class of double-stranded DNA insect viruses, their replication cycle produces two distinct types of virions: occlusion-derived virions (ODVs) and budded virions (BVs) [29]. ODVs initiate primary infection by specifically targeting midgut epithelial cells, whereas BVs mediate systemic dissemination through the haemocoel, enabling intercellular and intertissue transmission via their fusogenic envelope protein GP64 [30]. GP64 is a Class III viral fusion protein that resides in a metastable prefusion conformation and mediates the entry of BVs into diverse cell types [31,32]. During BV entry, endosomal acidification triggers a conformational shift in GP64 from its active prefusion to an inactive postfusion conformation, simultaneously mediating viral envelope–endosomal membrane fusion and pore formation to enable nucleocapsid release into the cytosol [30]. The baculovirus expression vector system (BEVS), developed based on the Autographa californica multiple nucleopolyhedrovirus (AcMNPV) BV, and by leveraging the cross-species infectivity mediated by GP64, is widely utilized in gene therapy, vaccine development, antibody production, and related fields [20,29,33,34,35,36,37].

Currently, the mainstream purification methods for BVs include affinity chromatography and centrifugation. Heparin affinity chromatography can concentrate viruses 200- to 700-fold, but the recovery rate of infectious viral particles is only about 26%, likely due to the disruption of the viral envelope during elution with high-salt buffers, which leads to viral inactivation [38,39]. Differential centrifugation combined with sucrose density gradient centrifugation is a traditional and effective technique for both concentration and purification of viral particles. However, the recovery rate of infectious virus particles is only 50% [40,41,42]. One reason for this could be the excessive centrifugal force, which over-compacts the pellet and damages the envelope, and the large fluctuations in gradient media concentration, which can cause osmotic pressure instability and disrupt the viral envelope [28]. In the purification methods of envelope proteins, the mainstream strategy generally involves expressing the target protein on the cell membrane system followed by its isolation and purification [26,43,44,45]. However, such methods typically fail to effectively preserve the protein’s active prefusion conformation.

To obtain high-quality samples with a high proportion of BV particles with intact envelopes, here, we combined conventional negative-staining transmission electron microscopy (TEM) with cryo-EM to compare the effects of discontinuous sucrose density gradient, continuous sucrose density gradient, and an optimized continuous sucrose density gradient on viral purity and percentage of BVs with intact envelopes. Our results indicate that the optimized continuous sucrose density gradient in this study not only increased the percentage of BV particles with intact envelopes from 36% to 81%, but also preserved the prefusion structure of GP64, thereby providing a reliable sample preparation method for future structural and functional studies of viral envelope proteins.

## 2. Materials and Methods

### 2.1. Cell Culture

Sf9 (*Spodoptera frugiperda*) cells were cultured in Sf-900 medium (Gibco, Carlsbad, CA, USA) supplemented with 10% fetal bovine serum (Excell, Shanghai, China) and 1% penicillin-streptomycin (Gibco) at 27 °C.

### 2.2. Construction, Passage, and Amplification of Recombinant Baculovirus

The transfer vector pFBDM-eGFP, containing the enhanced green fluorescent protein gene (*egfp*), was constructed using Gibson assembly (Invitrogen, Carlsbad, CA, California, USA). It was then transposed into *E. coli* DH10B (Invitrogen) containing Ac-Bacmid and a helper plasmid, and recombinant Bacmid was obtained through blue–white screening. Recombinant AcMNPV (rAcMNPV) was constructed using the Bac-to-Bac system, and virus amplification was performed as described in references [46]. The experimental procedure is briefly described as follows: Sf9 cells seeded in a 12-well plate were transfected with the recombinant Bacmid. After 7 days post-transfection, eGFP fluorescence was detected, and the supernatant was collected and designated as the P1-generation BV particles. Then, 15 µL of the P1-generation BV particles was used to infect 15 mL of suspension-cultured Sf9 cells. When the cell death rate exceeded 20% (approximately 6 days), the supernatant containing baculovirus was collected by centrifugation and designated as the P2-generation BV particles. The P2-generation BV particles were stored for subsequent use.

### 2.3. Differential Centrifugation

A quantity of 100 mL of suspension-cultured Sf9 cells at a cell density of 3 × 10^6^ cells/mL were inoculated with 10mL of P2-generation BVs for 48 h. After detecting eGFP fluorescence, the supernatant was collected by centrifugation at 4 °C, 1000× *g* for 10 min. The supernatant was further centrifuged at 4 °C, 10,000× *g* for 15 min to remove cell debris. The resulting supernatant was subjected to ultracentrifugation using an Optimality XPN-100 Ultracentrifuge (SW32 Ti rotor, Beckman, Brea, CA, USA) at 4 °C, 70,000× *g* for 45 min, and the pellet was resuspended in 100 µL phosphate-buffered saline (PBS, pH = 8, Millipore, Burlington, MA, USA).

### 2.4. Sucrose Density Gradient Centrifugation

Sucrose solutions of 10%, 20%, 30%, 40%, and 50% (*W*/*V*) were prepared. For the discontinuous gradients, 7 mL of each sucrose concentration was layered to create a 10–50% gradient. Continuous gradients of 10–60% (*W*/*V*) and 15–50% (*W*/*W*) sucrose were prepared using a Gradient Master (Biocomp, Victoria, BC, Canada).

An amount of 1 mL of the virus sample was added to the continuous and discontinuous sucrose density gradients, and ultracentrifugation was performed at 4 °C, 100,000× *g* for 2 h using an Optimality XPN-100 Ultracentrifuge (SW32 or SW41 Ti rotor, Beckman). The tip of a standard 200 μL micropipette was cut so that the tip mouth was approximately 0.3–0.4 cm wide. The modified tip was positioned along the tube wall immediately above the gradient interface, and the solution was collected until target fractions were obtained. Subsequently, the collected fractions were diluted with PBS, followed by sucrose removal through ultracentrifugation at 4 °C, 70,000× *g* for 45 min. The pellets were each resuspended in 100 µL TN buffer (50 mM Tris-HCl pH 8, 150 mM NaCl).

To minimize envelope damage, we optimized the continuous sucrose gradient protocol as follows: (1) increasing the volume of infected Sf9 cells (500 mL, 3 × 10^6^ cells/mL) to enhance BV yield; (2) employing a 15–50% (*W*/*W*) continuous sucrose gradient for homogeneous particle aggregation; (3) after centrifugation, the pellet was immersed in PBS overnight to loosen the compacted material prior to gentle resuspension, thus avoiding shear stress; (4) implementing gradual sucrose dilution to prevent osmotic shock. An amount of 5 to 10 μL of buffer was added each time, and the solution mixed. The above steps were repeated until the total volume of the solution reached 10 mL.

### 2.5. Negative-Staining Sample Preparation

Carbon-coated copper grids (Quantifoil, Jena, Germany) were hydrophilized by glow discharge at 35 mA for 1 min. A 3.5 µL sample was added to the grid, adsorbed for 30 s, washed twice with ddH_2_O, and stained with 2% (*W*/*V*) uranyl acetate. Samples were imaged using a TF20 TEM or Talos L120C (FEI, Hillsboro, OR, USA), and images were captured with a Tietz TemCam-F416 (TVIPS, Gauting, Germany) or Ceta CMOS camera (FEI).

### 2.6. Cryo-Electron Microscopy Sample Preparation and Examination

The Vitrobot Mark IV was set to 4 °C with 100% humidity, blot time of 3 s, and blot force of −5. A 3.5 µL sample was added to glow-discharged 300-mesh R1.2/1.3 or 300-mesh R3.5/1 Cu grids (Quantifoil), and rapidly frozen in liquid nitrogen. The frozen grids were transferred to cryo-EM sample holders, placed in a TF20 or Titan Krios G3i TEM (FEI), and images were acquired with a Tietz TemCam-F416 or K3 camera (Gatan, Pleasanton, CA, USA). For each sample, two random fields were recorded, and the percentage of BVs with intact envelopes was counted. Three biologically independent replicates were performed. Data were analyzed for significance with unpaired *t*-tests or one-way ANOVA (Welch).

### 2.7. GP64 Protein Purification

Purified BV particles were subjected to envelope protein purification using the mild detergent dodecyl maltoside (DDM) and cholesteryl hemi-succinate (CHS) (Anatrace, Maumee, OH, USA). The experimental procedure was as follows: The final concentration of 1% DDM and 0.1% CHS was prepared in TN buffer containing BV particles and incubated at 4 °C on a rotator for 2 h to solubilize the envelope protein. The solubilized proteins were purified using a chromatography system (ÄKTA pure, Marlborough, MA, USA) equipped with a Superdex 200 Increase 10/300 GL column (Cytiva, Marlborough, MA, USA). The mobile phase consisted of TN buffer containing 0.02% DDM and 0.002% CHS, with a flow rate set to 0.4 mL/min. The column was first washed with 25 mL of the mobile phase, followed by sample loading. Protein fractions were collected based on the A_280_ chromatographic peaks, concentrated to approximately 1 mg/mL, and used for cryo-EM sample preparation and data collection.

### 2.8. Western Blotting

An amount of 10 µL of each pre-concentrated protein fractions were mixed with Laemmli sample buffer (4×, BIO-RAD, Hercules, CA, USA) and incubated at 100 °C for 5 min. Equal amounts of protein were then loaded onto a 12% gel (BIO-RAD). After electrophoresis, the proteins were transferred to a PVDF membrane (BIO-RAD) and incubated overnight at 4 °C with the GP64 primary antibody AcV5 (Invitrogen). Subsequently, the membrane was incubated with secondary antibodies conjugated to horseradish peroxidase (HRP) (Thermo Scientific, Waltham, MA, USA), and images were captured using the ChemiDoc XRS + System (BIO-RAD) after chemiluminescence detection with Super Signal West Atto (Thermo Scientific).

### 2.9. Cryo-EM Data Collection and Processing

Cryo-EM data acquisition was achieved with serialEM (Boulder, CO, USA) [47], and performed using a 20 eV energy filter slit. Images were recorded at 130,000× magnification, corresponding to a pixel size of 0.33 Å/pixel in the super-resolution mode of the camera. A defocus range of −1.0 to −1.8 μm was set. A total dose of 50e^-^/Å2 at a dose rate of ~13e^-^/pixel/s was fractionated into 50 frames [48].

Cryo-EM data processing was performed with cryoSPARC (Toronto, ON, Canada) following regular single-particle procedures [49]. Patch motion correction was applied to the collected multi-frame data for motion correction, with the first two frames of each movie stack excluded during processing [50]. A binning factor of 2 was applied during motion correction, leading to a pixel size of 0.66 Å/pixel for the micrographs [51]. Contrast transfer function estimation was then performed to determine defocus and astigmatism parameters of the images. Protein particles were selected via blob picking and subjected to 2D classification. Particles lacking clear protein features were removed through 2D classification, and iterative refinement of the images was carried out using 2D averaging. The selected 2D particles were used for ab initio 3D reconstruction to generate a 3D model, which was subsequently refined through heterogeneous refinement. The model and protein structures were processed using UCSF Chimera (San Francisco, CA, USA), and the prefusion structure of GP64 (PDB code 8YG6) was downloaded from the Protein Data Bank (PDB) [32,52].

## 3. Results

### 3.1. Generation of Recombinant AcMNPV

**Figure 1:**
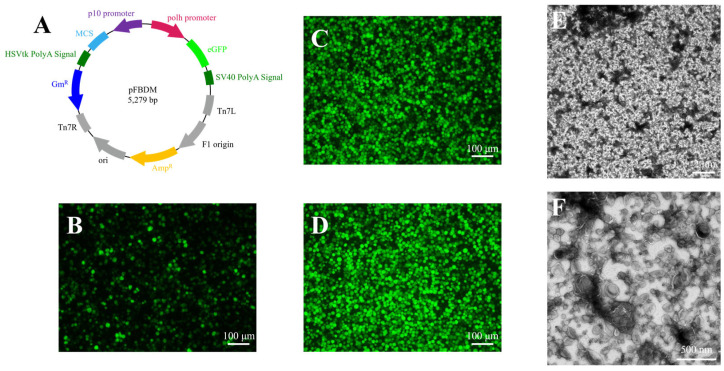
Construction, amplification, and crude purification of the recombinant baculovirus. (**A**) Schematic representation of the pF-polh-eGFP transfer vector. (**B–D**) Fluorescence images of Sf9 cells infected with P1, P2, and P3 generations of recombinant baculovirus. (**E**,**F**) The negative-staining TEM images show at 2300× magnification (**E**) and 16,000× magnification (**F**) of BV particles following differential centrifugation.

The transfer plasmid pFBDM-eGFP was generated by recombining the *egfp* gene downstream of the polyhedrin (*polh*) promoter in the pFBDM vector (Figure 1A). Recombinant baculovirus was engineered using the Bac-to-Bac system. Briefly, recombinant Ac-Bacmid was transfected into Sf9 cells. At six days post-transfection, eGFP expression was confirmed via fluorescence microscopy (Figure 1B), and the supernatant containing progeny virions was harvested as the P1-generation recombinant baculovirus. The P2-generation recombinant baculovirus stock was produced by infecting fresh Sf9 cells with the P1 viral stock (Figure 1C). The P2 baculovirus was then used to propagate rAcMNPV virions for downstream purification and characterization (Figure 1D).

To validate the composition of the viral sample, supernatants from P2 baculovirus-infected Sf9 cells were subjected to differential centrifugation for preliminary purification. Pelleted virions were resuspended in PBS and observed by negative-staining TEM. The results showed that the preliminary purified samples contained abundant vesicles, to the point that BV particles were barely visible (Figure 1E,F). The BV particles obtained from preliminary purification subsequently underwent additional purification through both discontinuous and continuous sucrose density gradient centrifugation methods, with a comparative analysis conducted to evaluate their respective purification efficiencies.

### 3.2. Purification of Recombinant AcMNPV via Discontinuous Sucrose Gradient Centrifugation

**Figure 2:**
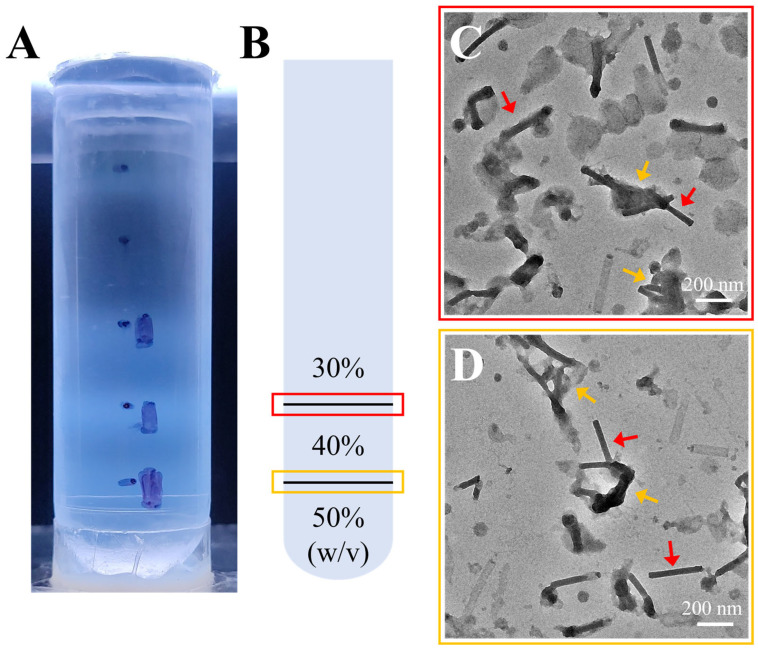
Sucrose density gradient purification and negative-staining TEM images. (**A**) The positions of bands formed in the centrifuge tubes after discontinuous sucrose density gradient centrifugation at concentrations of 10%, 20%, 30%, 40%, and 50% (*W*/*V*). (**B**) Diagram illustrating the band positions and sucrose density boundaries from (**A**), with 30%, 40%, and 50% representing sucrose densities. The black lines indicate the band positions, and the red and orange boxes correspond to panels (**C**) and (**D**), respectively. (**C**,**D**) Negative-staining TEM images of the two bands shown in (**B**), 11,500× magnification. The red arrows indicate BV nucleocapsids, and the orange arrows indicate the aggregated nucleocapsids.

Initially, 10–50% (*W*/*V*) discontinuous gradient centrifugation was employed to remove the extraneous debris and vesicles that were intermingled with the BV particles. Distinct light-scattering bands formed at sucrose layer interfaces spanning 20–50% (*W*/*V*) (Figure 2A). Bands corresponding to the 30%, and 40% sucrose layers were selectively harvested and designated as 30% sucrose and 40% sucrose fractions, respectively (Figure 2B). Collected fractions were diluted in PBS and centrifuged to remove sucrose, then resuspended in PBS for analysis by negative-stain TEM.

Negative-stain TEM analysis of sucrose gradient fractions revealed distinct particle compositions. The 30% sucrose fraction contained a heterogeneous population of vesicles and BV nucleocapsids (Figure 2C). The 40% sucrose fraction was dominated by BV nucleocapsids, though minor vesicular contaminants persisted (Figure 2D). Viral envelope integrity was notably disrupted during negative-staining, likely attributable to staining-induced dehydration artifacts. Due to the destruction of the envelope, only BV nucleocapsids could be observed in the images. The residual envelope fragments occasionally localized to one pole of the nucleocapsids, and aggregation of incomplete BV particles was frequently observed (orange arrows).

At the 30–40% (*W*/*V*) sucrose interface, the solution density increased from 1.127 g/mL to 1.176 g/mL, effectively removing the majority of vesicles. The banding of virions at the 40–50% (*W*/*V*) sucrose interface correlated with a density range of 1.176–1.230 g/mL. These results demonstrated that discontinuous sucrose density gradients (10–50% *W*/*V*) effectively purified and concentrated BV particles.

### 3.3. Purification of Recombinant AcMNPV via Continuous Sucrose Gradient Centrifugation

**Figure 3:**
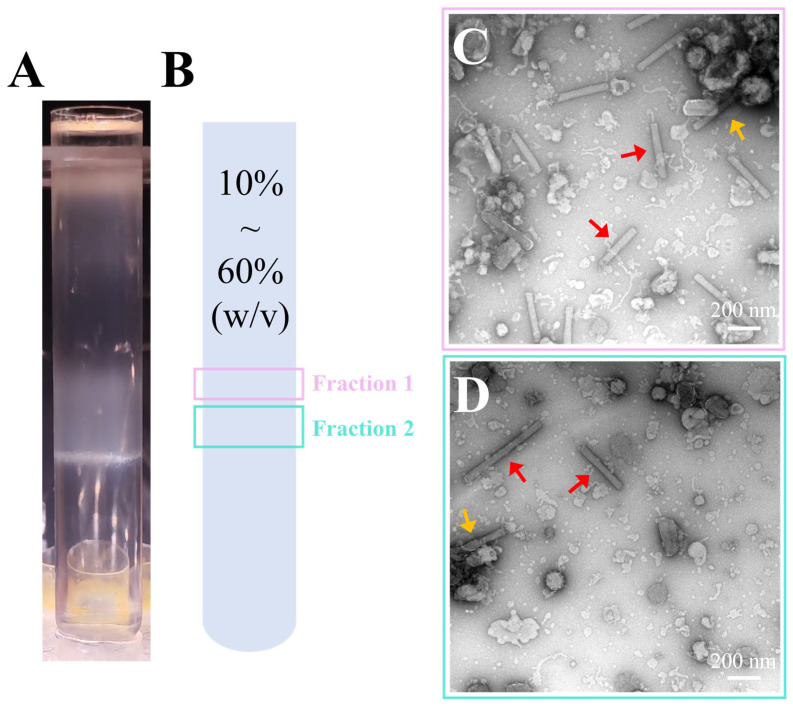
Continuous sucrose density gradient purification and negative-staining TEM images. (**A**) Positions of bands formed in the centrifuge tubes after continuous sucrose density gradient centrifugation at concentrations ranging from 10% to 60% (*W*/*V*). (**B**) Diagram illustrating the band positions from (**A**), with pink and turquoise boxes indicating Fraction 1 and Fraction 2, corresponding to panels (**C**) and (**D**), respectively. (**C**,**D**) Negative-staining TEM images of the two bands shown in (**B**), 11,500× magnification. The red arrows indicate BV nucleocapsids, and the orange arrows indicate the aggregated nucleocapsids.

Although discontinuous gradient centrifugation effectively removed the majority of superfluous debris and vesicles, this procedure still left residual impurities, highlighting a need for refinement in purification methodologies. To address these limitations, we adopted 10–60% (*W*/*V*) continuous gradient centrifugation to enhance the quality of purified BV. During centrifugation, multiple light-scattering bands were observed in the centrifuge tube (Figure 3A). The lowermost band, located near the tube’s base, consisted of flocculent material, while the two upper bands were isolated and designated as Fraction 1 (Frac 1) and Fraction 2 (Frac 2) (Figure 3B). These fractions were subsequently subjected to negative-staining TEM for detailed analysis.

Negative-stain TEM images showed that substrates in both bands were composed of BV particles (Figure 3C,D), but due to the negative-staining agent, only the naked nucleocapsids could be observed in the images (red arrow). Additionally, some aggregated nucleocapsids were also observed (orange arrow). Compared to Frac 2, Frac 1 exhibited a narrower band, indicating a more uniform morphology of virus particles (Figure 3A,B). This may have been due to the disruption of the viral envelopes during the purification process, which led to heterogeneity in particle morphologies and consequently the formation of two distinct bands. Additionally, vesicle contamination was significantly reduced in both bands, though some fragmented envelope-like materials were still observed. Due to the severe damage of viral envelopes by negative-staining reagents [53], the 40% sucrose sample from the discontinuous sucrose density gradient and the Frac1 sample from the continuous sucrose density gradient were further prepared as cryo-EM samples and re-evaluated for viral particle morphology.

**Figure 4:**
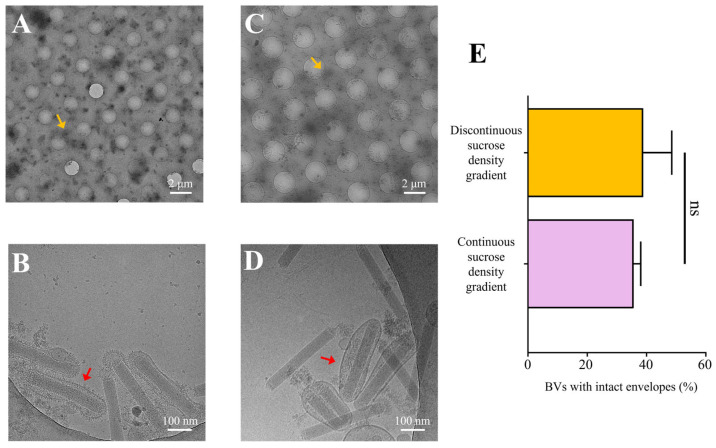
Cryo-EM samples of BV particles purified by sucrose density gradient centrifugation. (**A**,**C**) Vitrified ice images of BV particles frozen after discontinuous and continuous sucrose density gradient purification, respectively (800× magnification). The orange arrows indicate aggregated BV particles. (**B**,**D**) Images showing the morphology of BV particles from (**A**) and (**C**), respectively (25,000× magnification). The red arrows indicate BV particles. (**E**) Average percentage of complete virus particles observed in random field of view from cryo-EM samples of BV particles purified by both discontinuous and continuous sucrose density gradients. Error bars represent the SD from the mean of independent replicates (*n*  =  3), (ns stands for not significant).

Cryo-EM images showed that, at 800x magnification, virus particles were sparsely and unevenly distributed in the vitreous ice, with some aggregating (orange arrow), indicating a relatively low viral concentration (Figure 4A,C). At 25,000× magnification images, some virus particles were intact (red arrow), while others had lost their envelopes, leaving only naked nucleocapsids. Additionally, vesicle contamination was still observed in the discontinuous sucrose density gradient-purified sample (Figure 4B). In randomly collected fields from each sample, the proportion of BV particles with intact envelopes was 41% and 35% for the discontinuous and continuous sucrose density gradient-purified samples, respectively (Figure 4E). These findings indicated that the osmotic pressure changes caused by the 10% sucrose concentration difference in discontinuous gradient centrifugation did not damage the virion envelope. Furthermore, both continuous and discontinuous sucrose density gradient purification methods did not have a significant impact on the integrity of the envelope.

While both continuous and discontinuous gradient centrifugation methods were effective in eliminating the majority of superfluous debris and vesicles, a comparison revealed that continuous sucrose density gradient centrifugation yielded BV particles of superior purity. Furthermore, cryo-EM sample analysis facilitated a clearer visualization of certain viral particles wrapped within intact envelopes. It was important to note, though, that the BV particle concentration and the proportion of those with intact envelopes were comparatively low.

### 3.4. Optimized Purification Methodology for AcMNPV

Both continuous and discontinuous sucrose density gradient centrifugations effectively purified BV particles, but the overall proportion of intact envelopes was lower. Compared with the discontinuous method, the continuous sucrose density gradient efficiently removed vesicle contamination and resulted in higher-quality virus particles. Therefore, we further purified the BV particles by optimizing the continuous sucrose density gradient.

**Figure 5:**
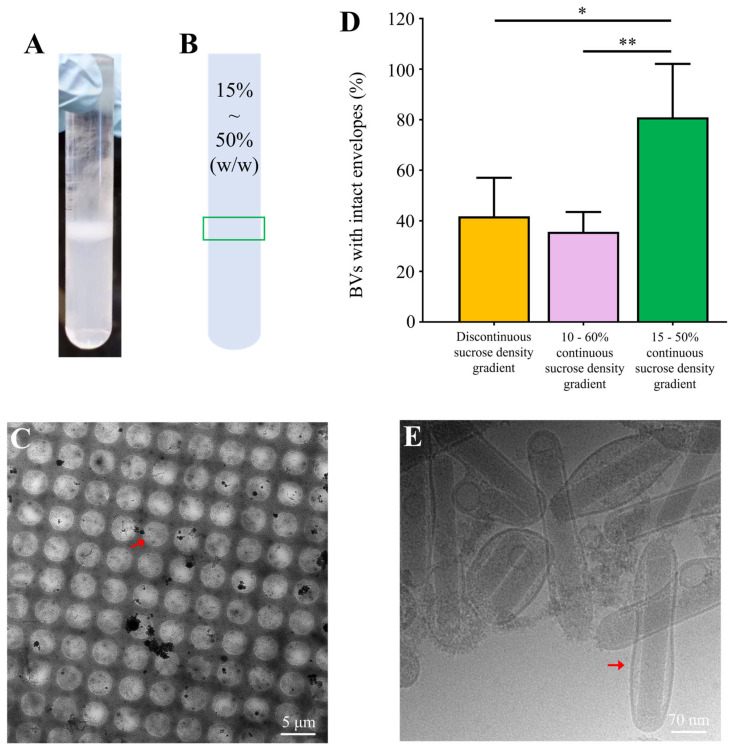
Optimized continuous sucrose gradient purification and cryo-EM analysis of intact virus particles. (**A**) Positions of the bands formed in the centrifuge tube after centrifugation of a continuous sucrose density gradient ranging from 10% to 50% (*W*/*W*). (**B**) Schematic representation of the band positions indicated in (**A**), with the band positions marked by the green box. (**C**) Vitrified ice image of the cryo-EM sample corresponding to the bands in (**B**), 800x magnification. The red arrows indicate uniformly distributed BV particles. (**D**) Proportion of intact BV particles in cryo-EM images after purification of discontinuous sucrose gradients of 10−50% (*W*/*V*), continuous sucrose gradients of 10−60% (*W*/*V*), and 15−50% (*W*/*W*). Error bars represent the SD from the mean of independent replicates (*n*  =  3), (* *p* < 0.05, ** *p* < 0.01). (**E**) Cyro-EM image of BV particles, 50,000× magnification. The red arrows indicate intact BV particles.

Following these optimizations, the resulting bands from the continuous sucrose density gradient centrifugation were more concentrated, with a single light-scattering band observed (Figure 5A,B), indicating a uniform viral particle population.

In cryo-EM images, 800× magnification images showed that most BV particles were uniformly distributed in the vitreous ice, with a substantial increase in virus density and a more even distribution (Figure 5C). The 50,000× magnification images showed that most virus particles had intact envelopes, and the morphology of the complete viral particles was clearly observable (Figure 5E). Statistical analysis of the proportion of BV particles with intact envelopes indicated that the proportion of intact envelopes doubled, with 80% of the viral particles retaining intact envelopes (Figure 5D). Compared to the unoptimized continuous sucrose density gradient, the optimized protocol improved the proportion of intact envelopes from 36% to 81%.

Although continuous density gradient centrifugation could yield high-purity BV particles, the proportion of intact envelopes was lower. Our optimized purification protocol enabled the isolation of high-purity BV particles, with over 80% of the viral particles retaining intact envelopes. This refined protocol provided high-quality samples for studies on envelope proteins.

### 3.5. Near-Native State of Metastable Prefusion GP64

The optimized continuous sucrose gradient protocol yielded high-quality BV particles with an intact envelope rate of 81%. To further assess the impact of this protocol on the conformation of the envelope protein GP64, we developed a novel strategy to isolate and purify the envelope protein from viral envelope (rather than from the cell membrane), and combined with cryo-EM 3D reconstruction to validate the GP64 conformation.

**Figure 6:**
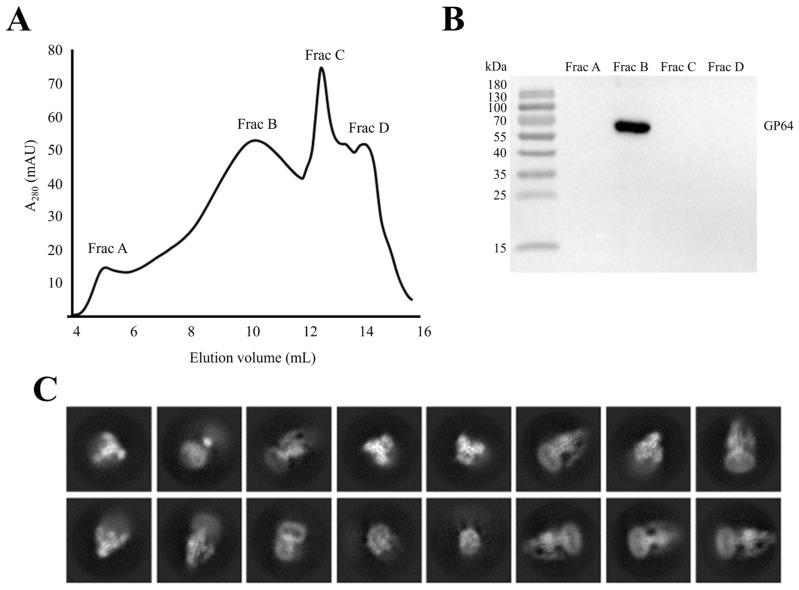
GP64 protein purification and cryo-EM data analysis. (**A**) Size-exclusion chromatography (SEC) profile of BV envelope. The X-axis represents elution volume in milliliters, while the Y-axis represents absorbance at 280 nm. Four representative peak fractions were marked. (**B**) Western blot analysis of the four peak fractions in the SEC profile. (**C**) Representative 2D class averages of particles for prefusion GP64.

Initially, membrane proteins were extracted from BV particles with high envelope integrity using a mild detergent (DDM: CHS), followed by purification via size-exclusion chromatography (SEC). Four distinct peaks were observed and designated as Frac A, Frac B, Frac C, and Frac D (Figure 6A). Western blot analysis of the four fractions using a GP64 monoclonal antibody (AcV5) confirmed that Frac B corresponded to the GP64 protein (Figure 6B).

**Figure 7:**
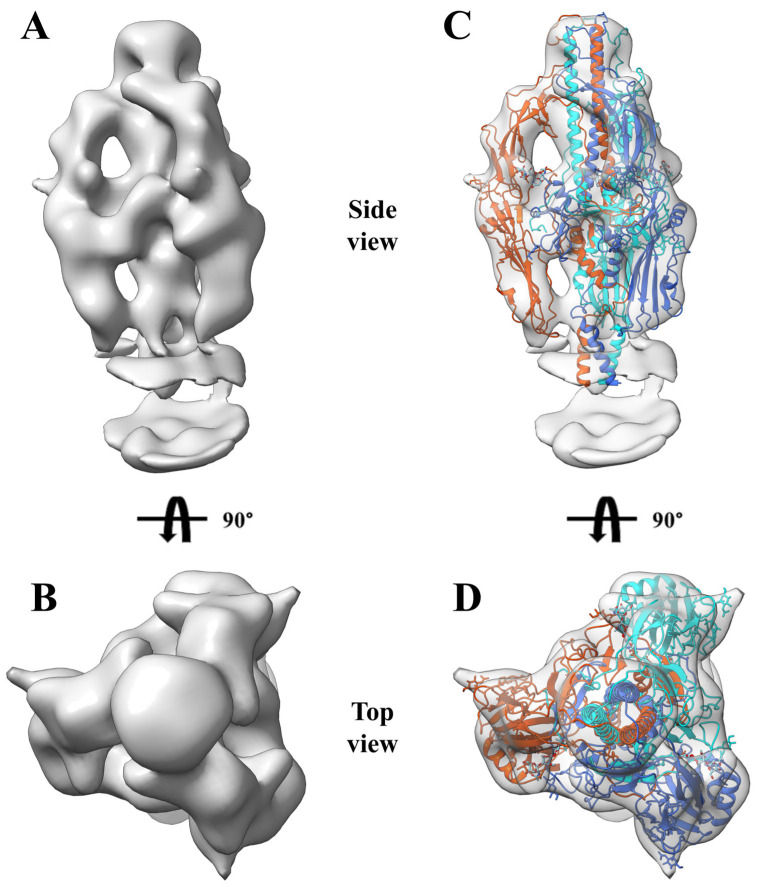
Cryo-EM density maps corresponding to prefusion GP64 (PDB 8YG6). (**A**,**B**) Side and top view of prefusion GP64 density map. (**C**,**D**) Side and top views showing the fit of prefusion GP64 (PDB 8YG6) with this density map. The three chains of prefusion GP64 are marked in dodger blue, orange red, and cyan, respectively.

Frac B was concentrated and prepared for cryo-EM analysis. After data acquisition, 49,001 particles were subjected to 2D classification in CryoSPARC. The 2D classification results showed well-defined protein structural features (Figure 6C). A total of 7913 particles exhibiting well-defined protein structural features were selected for 3D classification, yielding a model with a resolution of 15 Å (Figure 7A,B). The prefusion GP64 structure (PDB 8YG6) was fitted into the density maps of the reconstructed model, revealing full compatibility between the prefusion conformation and the 3D reconstruction (Figure 7C,D), confirming that the purified GP64 retained its metastable prefusion conformation.

The optimized continuous sucrose gradient protocol not only preserved the envelope integrity of BV particles but also maintained the native metastable prefusion conformation of GP64. This finding not only validated the reliability of our methodology for purifying metastable envelope proteins but also provided a novel strategy for isolating other prefusion conformation envelope proteins whose structures remain unresolved.

## 4. Discussion

In this study, we employed cryo-EM to evaluate the impact of different purification methods on the proportion of intact envelopes of BV particles and demonstrated that the optimized purification protocol can preserve the prefusion state of the envelope protein GP64. Following preliminary purification by differential centrifugation, the BV particles were further purified using discontinuous sucrose density gradient, continuous sucrose density gradient, and an optimized continuous sucrose density gradient developed in this study. The results indicated that both the continuous and discontinuous sucrose density gradient centrifugation methods primarily influenced viral purity without significantly affecting the integrity of the viral particles. Notably, through optimizations such as reducing shear force during resuspension and gradually diluting high-concentration sucrose to minimize osmotic pressure on the envelope, the proportion of intact envelopes was successfully increased. After optimizing the continuous sucrose density gradient protocol, the proportion of virus particles with intact envelopes increased from 36% to 81%, significantly preserving the native morphology of the virus particles. Moreover, the strategy of directly purifying envelope proteins from the viral envelope successfully maintained the active prefusion conformation of GP64.

In the cryo-EM samples, intact BV particles exhibited a distinctive “bipolar anchoring” structure, whereby the two ends of the rod-shaped nucleocapsid appeared to be connected to the viral envelope with specific protein interactions (Figure 5E). Additionally, even some virus particles with damaged envelopes retained residual envelope fragments at both ends (Figure 4B,D). In contrast, the lateral aspects of the nucleocapsid showed no apparent interaction with the envelope, resulting in the formation of an intermembrane gap that occasionally contained small vesicles (Figure 5E). This unique structural feature suggested that mechanical shear forces during resuspension preferentially disrupted envelope–nucleocapsid interactions at the lateral regions, supporting the need for our gentle handling approach.

Compared to the GP64 crystal structure obtained by X-ray crystallography, the structure resolved by cryo-EM more closely resembled the in situ conformational state of the protein. In the field of envelope protein purification, conventional experimental strategies typically entail expressing the target protein on the cell biological membrane system and subsequently isolating it through detergent treatment [32,45]. However, this expression system has significant limitations: compared to the in situ envelope proteins in the virus, heterologously expressed envelope proteins not only lose their native microenvironment but are also more prone to conformational changes due to their metastable nature [25,44]. To maintain the biologically active prefusion conformation, current methods generally require site-directed mutagenesis of flexible protein domains or employ antibody/small-molecule complexes for conformational stabilization [26,43]. The strategy employed in this study, which directly isolates GP64 from the viral envelope, circumvented the unavoidable steps in traditional envelope protein isolation methods such as cell lysis, sonication, and mechanical shear forces. This novel purification strategy not only maximally preserved the native structural features of the protein but also provided a reliable technical foundation for precisely analyzing the functional conformations of envelope proteins.

Despite these significant advancements, our study had certain limitations. First, approximately 19% of the virus particles still lost their envelope suggesting that further improvements were possible; future investigations could explore novel gradient media (e.g., potassium tartrate–glycerol) to further enhance envelope integrity [54]. Second, the continuous sucrose density gradient method might not be suitable for large-scale purification of baculovirus, so selecting an appropriate purification method based on specific application requirements is critical.

From an application standpoint, our findings hold dual value. On one hand, high-integrity virus samples provide an ideal substrate for elucidating the native conformation of envelope proteins. Since envelope proteins are key mediators of viral entry, structural characterization of their prefusion conformations could drive the development of novel antiviral drugs [55,56,57,58]. On the other hand, the purification protocol described here offers an important reference for the preparation of other enveloped viruses (e.g., Herpes simplex virus, Epstein–Barr virus). Considering that over 60% of FDA-approved drugs target membrane proteins [59], and that conventional membrane protein purification methods often result in structural distortion requiring additional stabilization strategies [43,60], the in situ purification strategy established in this study opens up a new avenue for obtaining membrane proteins in their near-native state.

## 5. Conclusions

In summary, the optimized purification protocol developed in this study effectively enhanced both the purity and envelope integrity of BV particles while preserving the prefusion conformation of GP64. This study provided a reliable sample preparation protocol for subsequent structural and functional studies. Future research could focus on further refining the purification process to obtain even higher-quality virus samples, thereby advancing virological research and its related applications.

## Data Availability

The original contributions presented in this study are included in the article/Supplementary Material. Further inquiries can be directed to the corresponding author.

## Supplementary Materials

The following supporting information can be downloaded at: https://www.mdpi.com/article/10.3390/insects16040424/s1, Figure S1: The original fluorescence microscope images in the manuscript (corresponding to Figure 1B−D in the manuscript); Figure S2−S4. The original negative-stained TEM images in the manuscript (corresponding to Figure 1E,F, Figure 2C,D, Figure 3C,D, and Figure 4A−D in the manuscript); Figure S5: The original cryo-EM images in the manuscript (corresponding to Figure 5C,E in the manuscript); Figure S6: The western blot original images in the manuscript (corresponding to Figure 6B in the manuscript); Table S1: Primer names and sequences for Gibson assembly (corresponding to plasmid pFBDM-eGFP); Table S2: Proportion of intact envelopes in six fields across different fractions after centrifugation in the manuscript (corresponding to Figure 4E and Figure 6B in the manuscript).

## Abbreviations

The following abbreviations are used in this manuscript:

AcMNPV	Autographa californica multiple nucleopolyhedrovirus
Cryo-EM	Cryo-electron microscopy
NPV	Nucleopolyhedrovirus
BEVS	Baculovirus expression vector system
TEM	Transmission electron microscopy
Sf9	*Spodoptera frugiperda* 9 cells
eGFP	Enhanced green fluorescent protein
rAcMNPV	Recombinant Autographa californica multiple nucleopolyhedrovirus
PBS	Phosphate-buffered saline
*W*/*V*	Weight/Volume
*W*/*W*	Weight/Weight
*polh*	Polyhedrin promoter
DDM	Dodecyl maltoside
CHS	Cholesteryl hemi-succinate
SEC	Size-exclusion chromatography
PDB	Protein Data Bank
