# Supplementary material for: Centrifugation-Based Purification Protocol Optimization Enhances Structural Preservation of Nucleopolyhedrovirus Budded Virion Envelopes
Source: Insects. 2025 Apr 17;16(4):424. doi: 10.3390/insects16040424 (PMC12027964; doi:10.3390/insects16040424)
Supplement: Supplementary file 1 [file insects-16-00424-s001.zip › insects-3566289-supplementary.pdf]

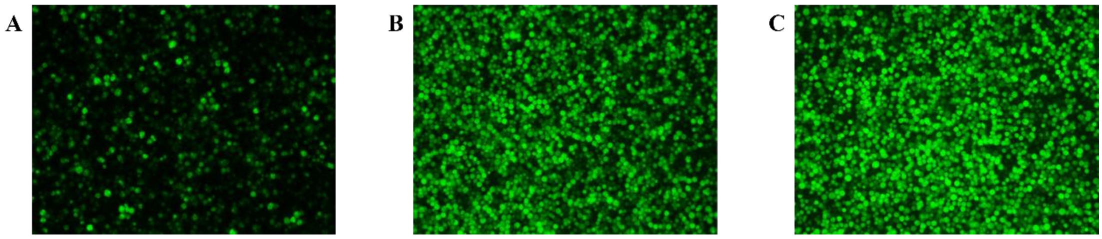

Figure S1. The original fluorescence microscope images in the manuscript. (A) Fluorescence images of Sf9 cells infected with the P1 generation of recombinant baculovirus (corresponding to Fig. 1B in the manuscript). (B) Fluorescence images of Sf9 cells infected with the P2 generation of recombinant baculovirus (corresponding to Fig. 1C in the manuscript). (C) Fluorescence images of Sf9 cells infected with the P3 generation of recombinant baculovirus (corresponding to Fig. 1D in the manuscript).

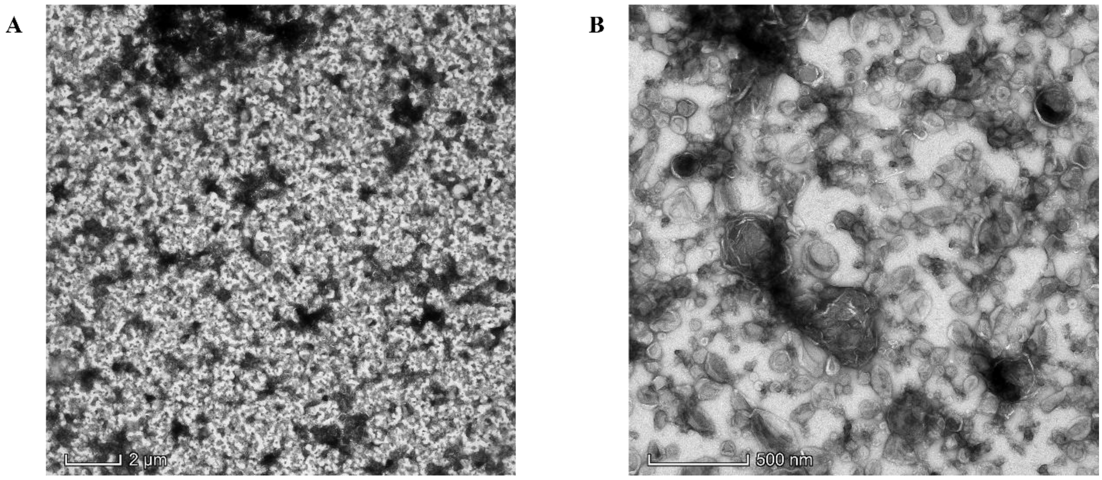

Figure S2. The original negative-stained TEM images of BV particles after differential centrifugation in the manuscript. (A) Image under 2,300x magnification (corresponding to Fig. 1E in the manuscript). (B) Image under 16,000x magnification (corresponding to Fig. 1F in the manuscript).

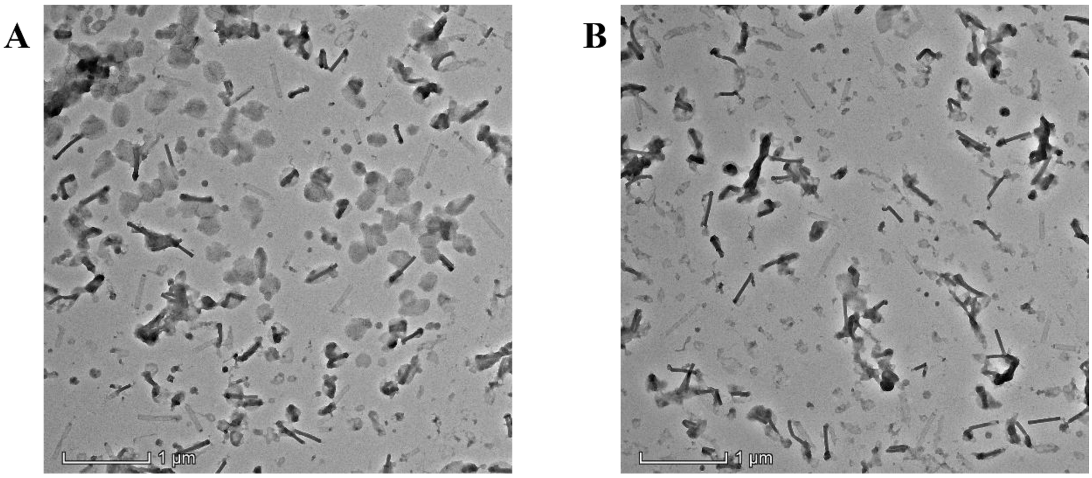

Figure S3. The original negative-stained TEM images of BV particles after discontinuous sucrose density gradient centrifugation in the manuscript. (A) Original images of the *30% sucrose* fraction (corresponding to Fig. 2C in the manuscript). (B) Original images of the *40% sucrose* fraction (corresponding to Fig. 2D in the manuscript).

A

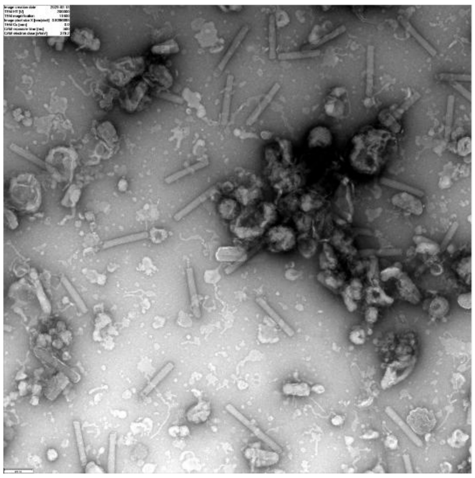

B

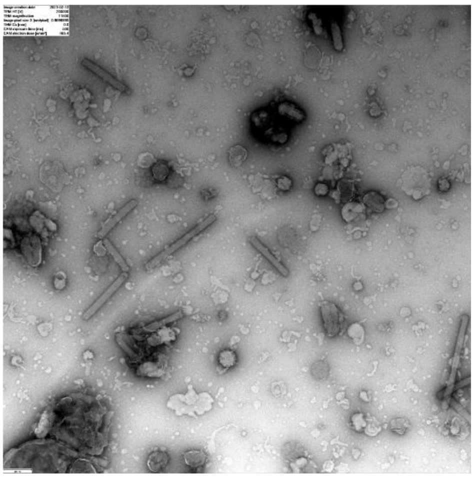

Figure S4. The original negative-stained TEM images of BV particles after continuous sucrose density gradient centrifugation in the manuscript. (A) Original images of Fraction 1 in the continuous sucrose density gradient (corresponding to Fig. 3C in the manuscript). (B) Original images of Fraction 2 in the continuous sucrose density gradient (corresponding to Fig. 3D in the manuscript).

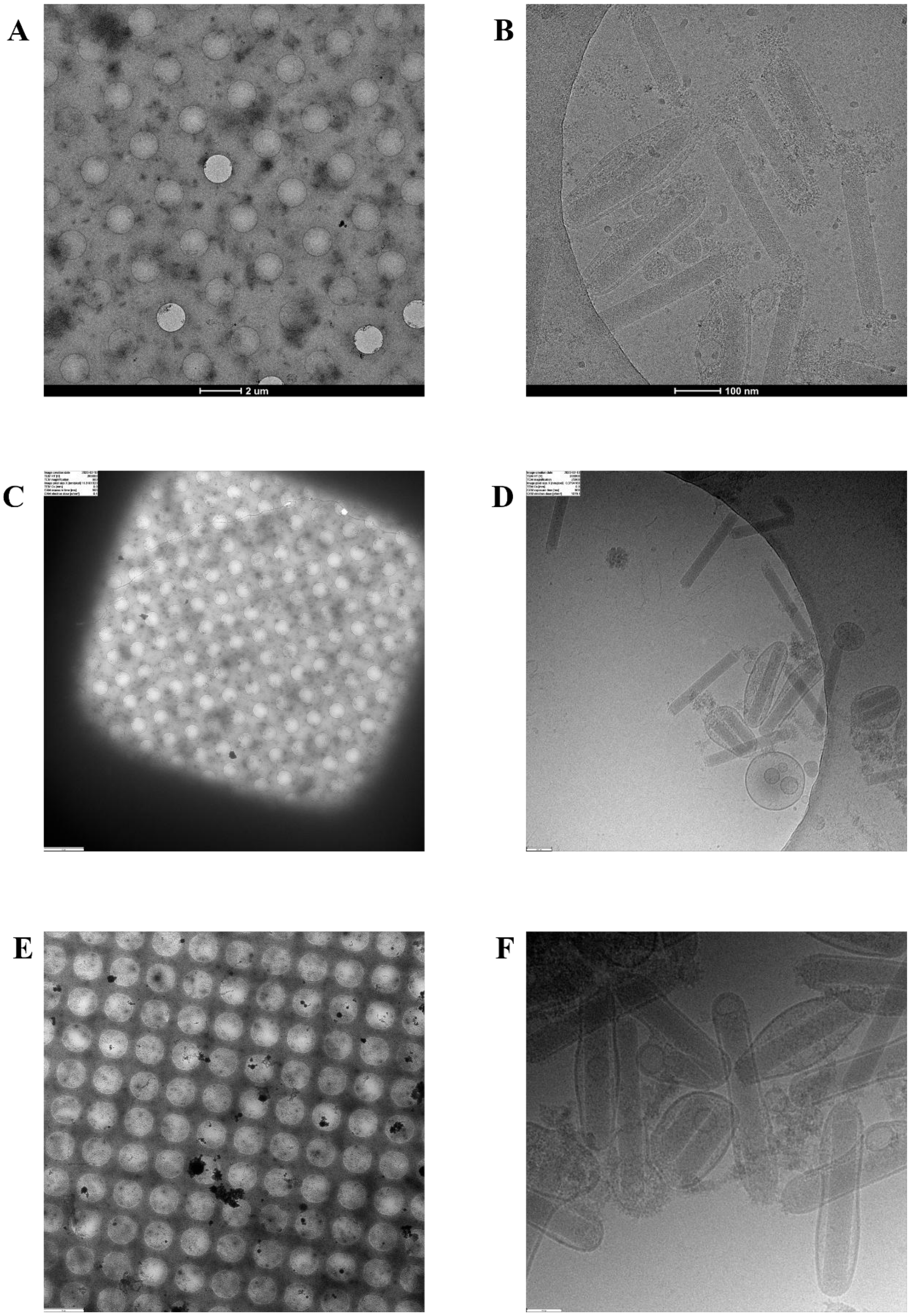

Figure S5. The original cryo-EM images in the manuscript. (A, B) Original images of the *40% sucrose* fraction (corresponding to Fig. 4A, B in the manuscript). (C, D) Original images of the Fraction 1 (corresponding to Fig. 4C, D in the manuscript). (E, F) Original images of the fraction from the optimized continuous sucrose density gradient (corresponding to Fig. 5C, E in the manuscript).

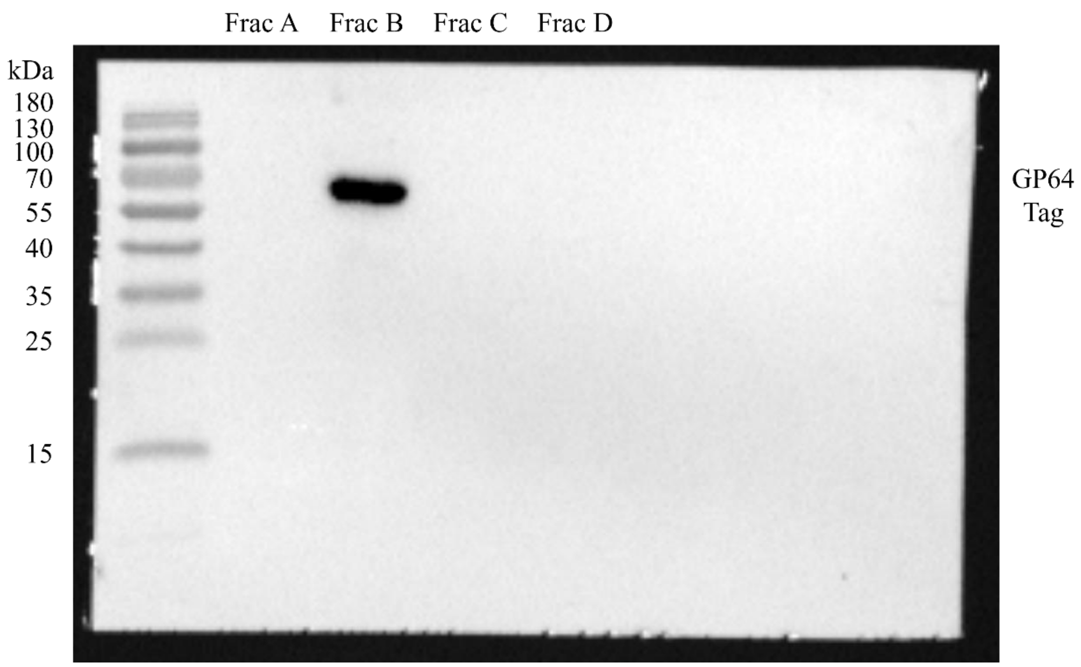

Figure S6. Original Western Blot images corresponding to the four peak fractions in the SEC profile (corresponding to Fig. 6B in the manuscript).

Table S1. Primer names and sequences for Gibson assembly (corresponding to plasmid pFBDM-eGFP).

Primer Name	Gene Name	Primer Sequences (5′–3′)
G-pF-F	eGFP	ccaccatcgggcgcggatccatggtgagcaagggcgagga
G-pF-R		gagctcgtcgacgtaggcctttacttgtacagctcgtcca
pF-G-F	pFBDM	tggacgagctgtacaagtaaaggcctacgtcgacgagctc
pF-G-R		tcctcgcccttgctcaccatggatccgcgcccgatggtgg

Table S2: Proportion of intact envelopes in six fields across different fractions after sucrose density gradient centrifugation (corresponding to Fig. 4E and Fig. 6B in the manuscript).

	Discontinuous sucrose density gradient	Continuous sucrose density gradient	Optimized continuous sucrose density gradient
Total number of virions 1	2	22	6
Total number of virions in the intact envelope 1	1	11	3
The proportion of intact envelop 1	50.00%	50.00%	50.00%
Total number of virions 2	7	15	8
Total number of virions in the intact envelope 2	3	4	8
The proportion of intact envelop 2	42.86%	26.67%	100.00%
Total number of virions 3	8	11	10
Total number of virions in the intact envelope 3	3	4	6
The proportion of intact envelop 3	37.50%	36.36%	60.00%
Total number of virions 4	3	12	7
Total number of virions in the intact envelope 4	2	4	7
The proportion of intact envelop 4	66.67%	33.33%	100.00%
Total number of virions 5	4	22	7
Total number of virions in the intact envelope 5	1	7	6
The proportion of intact envelop 5	25.00%	31.82%	85.71%
Total number of virions 6	7	28	10
Total number of virions in the intact envelope 6	2	10	9
The proportion of intact envelop 6	28.57%	35.71%	90.00%
